# Comparing model‐based cerebrovascular physiomarkers with DTI biomarkers in MCI patients

**DOI:** 10.1002/brb3.1356

**Published:** 2019-07-09

**Authors:** Vasilis Z. Marmarelis, Dae C. Shin, Takashi Tarumi, Rong Zhang

**Affiliations:** ^1^ Department of Biomedical Engineering University of Southern California Los Angeles California; ^2^ Biomedical Simulations Resource Center University of Southern California Los Angeles California; ^3^ Neurology and Neurotherapeutics UT Southwestern Medical Center Dallas Texas; ^4^ Institute for Exercise and Environmental Medicine Texas Health Presbyterian Hospital Dallas Texas; ^5^ Present address: Human Informatics Research Institute National Institute of Advanced Industrial Science and Technology Tsukuba Japan

**Keywords:** Alzheimer's disease, cerebral flow regulation, diffusion tensor imaging, hemodynamic physiomarkers, mild cognitive impairment, model‐based diagnostic physiomarkers

## Abstract

**Objective:**

To compare the novel model‐based hemodynamic physiomarker of Dynamic Vasomotor Reactivity (DVR) with biomarkers based on Diffusion Tensor Imaging (DTI) and some widely used neurocognitive scores in terms of their ability to delineate patients with amnestic Mild Cognitive Impairment (MCI) from age‐matched cognitively normal controls.

**Materials & Methods:**

The model‐based DVR and MRI‐based DTI markers were obtained from 36 patients with amnestic MCI and 16 age‐matched controls without cognitive impairment, for whom widely used neurocognitive scores were available. These markers and scores were subsequently compared in terms of statistical delineation between patients and controls.

**Results:**

It was found that statistically significant delineation between MCI patients and controls was comparable for DVR or DTI markers (*p* < 0.01). The performance of both types of markers was consistent with the scores of some (but not all) widely used neurocognitive tests.

**Conclusion:**

Since DTI offers a measure of cerebral white matter integrity, the results suggest that the model‐based hemodynamic marker of DVR may correlate with cognitive impairment due to white matter lesions. This finding is consistent with the hypothesis that dysregulation of cerebral microcirculation may be an early cause of cognitive impairment, which has been recently corroborated by several studies.

## 1 INTRODUCTION

The quest for noninvasive diagnostic markers of early‐stage Alzheimer's disease (AD) has led to the development of biomarkers derived from Magnetic Resonance Imaging (MRI) and Diffusion Tensor Imaging (DTI), in addition to the traditional neurocognitive testing scores. Individuals with amnestic Mild Cognitive Impairment (MCI) have high risk of developing AD, and MCI has been considered a transitional stage between normal aging and AD. Recently, model‐based “physiomarkers” of cerebral hemodynamics have also been introduced for the same purpose under the hypothesis that dysregulation of cerebral microcirculation may cause cognitive impairment (Marmarelis, Shin, Orme, & Zhang, 2013; Marmarelis, Shin, Tarumi, & Zhang, 2017). The use of MRI and DTI markers is based on the premise that the onset of neurodegenerative disease is associated with an elevated risk for structural and functional abnormalities in the brain that are detectable by MRI and/or DTI (Charlton et al., 2006; Madden, Bennett, & Song, 2009; Mori & Zhang, 2006; Sexton, Kalu, Filippini, Mackay, & Ebmeier, 2011; Smith et al., 2006; Sun et al., 2006; Tarumi et al., 2015; Wardlaw et al., 2013). Much attention has been accorded to the presence of white matter lesions as an important risk factor for cognitive impairment (Groot et al., 2013; Maillard et al., 2014; Young, Halliday, & Kril, 2008). Although markers based on structural MRI have some utility, more promise has been offered by DTI‐based biomarkers that quantify the neuronal fiber integrity in white matter using measures such as Fractional Anisotropy (FA) and Radial or Mean Diffusivity (RD or MD) (Beaulieu & Allen, 1994; Mori & Zhang, 2006). Correlation between these DTI‐based markers and neurocognitive performance has been demonstrated (Madden et al., 2009). Specifically, these DTI‐based biomarkers have been shown to correlate with the myelination level and the density of the neuronal fibers (Song et al., 2003). Since the main cause of these white matter lesions may be rooted in abnormalities of cerebral blood circulation and cardiovascular regulation, leading to cerebral hypoperfusion or ischemia (Fernando et al., 2006; Moody, Bell, & Challa, 1990), we wish to explore possible correlations between these DTI‐based biomarkers and model‐based physiomarkers of cerebral hemodynamics that we have recently introduced (Marmarelis et al., 2017) in order to delineate MCI patients from age‐matched controls without cognitive impairment. Our working hypothesis is that if white matter lesions are associated with abnormalities in cerebral microcirculation, then our cerebral hemodynamic physiomarkers may correlate with the DTI‐based biomarkers of cerebral white matter neuronal fiber integrity.

## 2 MATERIALS AND METHODS

### 2.1 Study participants

This study enrolled 52 participants, 36 patients with amnestic MCI (19 female and 17 male), and 16 age‐matched cognitively normal controls (eight female and eight male) through a community‐based advertisement and the University of Texas Southwestern Medical Center Alzheimer's Disease Center. Inclusion criteria were as follows: men and women aged 55–80 years with normal cognitive function or MCI. Exclusion criteria included a history of cardiovascular (e.g., angina, myocardial infarction), cerebrovascular, or psychiatric disease, uncontrolled hypertension or dyslipidemia, diabetes mellitus, obesity (body mass index >35 kg/m^2^), current or a history of smoking within the past 2 years, or chronic inflammatory disease. Individuals with a pacemaker or any metal in their body that precludes MRI were also excluded. The diagnosis of amnestic MCI was based on the Petersen criteria (Petersen et al., 1999), as modified by the Alzheimer's Disease Neuroimaging Initiative project. Clinical evaluation was based on the recommendations of the Alzheimer's Disease Cooperative Study (Morris et al., 1997). Specifically, MCI patients had to meet the following criteria: (a) subjective memory complaint, (b) a global Clinical Dementia Rating of 0.5 with a score of 0.5 in the memory category, (c) objective memory loss as indicated by the Logical Memory subtest of the Wechsler Memory Scale‐Revised, and (d) Mini‐Mental State Exam score between 24 and 30. All subjects signed the informed consent form approved by the Institutional Review Boards of the University of Texas Southwestern Medical Center and the Texas Health Presbyterian Hospital of Dallas.

### 2.2 Diffusion tensor imaging

A 3‐T magnetic resonance imaging **(**MRI) scanner (Philips Medical System) was used to acquire the Diffusion Tensor Imaging (DTI) data using a single‐shot echo planar imaging (EPI) sequence with a sensitivity encoding (SENSE) parallel imaging scheme (reduction factor = 2.2). The imaging matrix was 112 × 112 with field of view (FOV) = 224 × 224 mm^2^ (nominal resolution of 2 mm), which was filled to 256 × 256 pixels. Axial slices of 2.2 mm thickness (no gap) were acquired parallel to the anterior–posterior commissure line. A total of 65 slices covered the entire hemisphere and brainstem. Echo time (TE)/repetition time (TR) was 51/5,630 ms. The diffusion weighing was encoded along 30 independent orientations, and the *b* value was 1,000 s/mm^2^. The scan duration was 4.3 min. Automated image registration was performed on the raw diffusion images to correct distortions caused by motion artifacts or eddy currents. The DTI scan was performed twice. For image preprocessing and voxel‐wise tract‐based spatial statistical (TBSS) analysis (Smith et al., 2004, 2006), we used the broadly accepted software library of FMRIB (FSL, https://surfer.nmr.mgh.harvard.edu/fswiki). The ROI analysis was performed using the *ICBM‐DTI‐81 white matter* atlas (Mori et al., 2008).

### 2.3 Neurocognitive assessment

For attention‐executive function assessment, this study used the following four tests: Trails‐making part B (Trails‐B), Wechsler Adult Intelligence Scale for digit span backward (WAIS‐dig), Wisconsin Card Sorting Test perceptive responses (WCST‐per), sum of Letter Fluency parts F, A and S (LF‐F&A&S) (Drane, Yuspeh, Huthwaite, & Klingler, 2002; Wechsler, 1987). For episodic memory assessment, this study used the following three tests: California Verbal Learning Test with long delay free recall (CVLT‐LDFR), California Verbal Learning Test total of parts 1–5 (CVLT‐total) (Delis, Kramer, Kaplan, & Ober, 2000), and Visual Reproduction t‐score recall (VRT).

### 2.4 Model‐based analysis of cerebral hemodynamic data

Cerebral hemodynamic time‐series data were collected at the resting state over 5–6 min (after 20 min of rest) via transcranial Doppler (TCD) at an initial sampling rate of 1 KHz of blood flow velocity (CBFV) at the middle cerebral arteries using a 2 MHz TCD probe (Multiflow, DWL) placed over the temporal window and fixed at constant angle with a custom‐made holder. Concurrently, continuous measurements of arterial blood pressure (ABP) were made with finger photo‐plethysmography (Finapres) and of end‐tidal CO2 (ETCO2) via a nasal cannula using capnography (Criticare Systems). All measurements are noninvasive, safe, and comfortable for the subjects. The data were collected in a quiet, environmentally controlled laboratory under resting seated conditions. The raw data were reduced to beat‐to‐beat values following our established preprocessing procedures (Marmarelis et al., 2017). The resulting beat‐to‐beat time‐series data were used to estimate predictive dynamic models following our novel modeling methodology (Marmarelis et al., 2017). The obtained predictive input–output models were then used to compute the predicted CBFV response of each subject to a 5‐s pulse input of ETCO2, while the other input (ABP) is kept at baseline. We have found in our previous work that the average of this model‐predicted CBFV response over 5 s represents a differentiating “physiomarker” for MCI patients (relative to age‐matched controls), which is termed “Dynamic Vasomotor Reactivity” (DVR) index, because it quantifies the cerebral flow response of each subject to a sudden (and short) change in ETCO2 (a surrogate for blood CO2 tension) (Marmarelis et al., 2017). We note that the DVR is distinct from conventional measures of cerebral vasoreactivity that are obtained with breath‐holding or CO2 inhalation. Detailed on this modeling methodology and its application to cerebral hemodynamics can be found in ref. (Marmarelis, Mitsis, Shin, & Zhang, 2016; Marmarelis, Shin, Orme, & Zhang, 2014; Marmarelis et al., 2017).

### 2.5 Statistical analysis

Our statistical analysis considered 11 different diagnostic markers or neurocognitive scores: the DVR, three DTI markers (FA, MD, and RD), four neurocognitive test scores for assessment of attention‐executive function (Trails‐B, WAIS‐dig, WCST‐per, LF‐F&A&S), and three neurocognitive test scores for assessment of episodic memory (CVLT‐LDFR, CVLT‐total, and VRT), which are described above. The ability of each marker/score to differentiate between the MCI patients and the cognitively normal controls was first assessed with the *t*‐statistic (*p*‐value). The Pearson pairwise correlations were also examined. Finally, a linear fixed effects statistical regression model was used to separate the effects of age, gender, and education (viewed as covariates) and subsequently examine the ability of the resulting markers/scores to differentiate between the MCI patients and the cognitively normal controls, as well as the possible linear pairwise relations that are revealed by linear regression lines. Statistical significance was set at *p* < 0.05. Statistical analysis was performed using MATLAB (MathWorks Inc.).

## 3 RESULTS

### 3.1 Group difference between patients and controls for each marker/score

Table 1 shows demographics of control subjects (CS) and MCI patients (MP). Table 2 shows the mean (*SD*) values for each of the 11 selected markers/scores and the *p*‐value for the statistical differentiation between the groups of 16 CS and 36 MP, *before and after* separating out the effects of the covariates (age, gender, and education). It is seen that 6 of the 11 markers/scores have *p* < 0.01 and one has *p* < 0.05. One DTI marker (FA) and three neurocognitive scores (WCST‐per, LF‐F&A&S, and VRT) do not allow statistically significant differentiation between MP and CS (*p* > 0.05) before separating the covariates effects, while only two neurocognitive scores (WCST‐per and LF‐F&A&S) do not allow statistically significant differentiation after separating the covariates effects.

**Table 1:** Participant demographics

	Normal (*N* = 16)	MCI (*N* = 36)	*p*‐value
	Mean ± *SD*	Mean ± *SD*	
Clinical dementia rating scale	0	0.5	
Men/Women (*n*)	8/8	17/19	
Age (years)	65 ± 7	65 ± 7	0.956
Education (years)	17 ± 2	16 ± 2	0.210
Height (cm)	172 ± 10	168 ± 9	0.241
Body mass (kg)	79 ± 17	79 ± 14	0.898
Body mass index (kg/m^2^)	26 ± 4	28 ± 4	0.269
Cardiovascular measurements			
24‐hr heart rate (bpm)	70 ± 7	70 ± 10	0.998
24‐hr systolic blood pressure (mmHg)	130 ± 11	132 ± 11	0.585
24‐hr diastolic blood pressure (mmHg)	75 ± 9	74 ± 8	0.822
Antihypertensive medication use (*n*, %)	9 (56%)	14 (39%)	0.245
Cholesterol medication use (*n*, %)	7 (44%)	8 (22%)	0.114
Cognitive screening scores			
Mini‐Mental State Exam score	29.2 ± 0.8	28.8 ± 1.5	0.316
Montreal cognitive assessment score	27.6 ± 1.9	24.6 ± 2.9	**<0.001**
Immediate logical memory score	14.6 ± 2.9	10.7 ± 2.3	**<0.001**
Long delayed logical memory score	14.4 ± 2.6	8.6 ± 2.0	**<0.001**
Brain volumetric measures			
Global brain volume (%ICV)	69.2 ± 3.9	69.6 ± 3.5	0.662
Hippocampus volume (%ICV)	0.518 ± 0.060	0.529 ± 0.063	0.569
White matter hyperintensity volume (%ICV)	0.110 ± 0.100	0.094 ± 0.105	0.634

Note

*p*‐values are based on independent *t* test or chi‐square test. *p* < 0.05 are bolded. Twenty‐four‐hour measurements are based on ambulatory blood pressure monitoring.

Abbreviations: ICV, intracranial volume; MCI, Mild Cognitive Impairment.

**Table 2:** Mean (*SD*) values of each marker/score and its *p*‐value for differentiation between the groups of 16 cognitively normal control subjects (CS) and of 36 MCI patients (MP) before and after covariate analysis

Markers/Scores		Before covariate analysis			After covariate analysis		
		Mean (*SD*) of 16 CS	Mean (*SD*) of 36 MP	*p*‐values	Mean (*SD*) of 16 CS	Mean (*SD*) of 36 MP	*p*‐values
1	DVR	1.1546 (0.6461)	0.5449 (0.6031)	**0.0034**	1.1546 (0.5789)	0.5457 (0.5825)	**0.0015**
2	FA	0.5950 (0.0283)	0.5772 (0.0327)	0.0546	0.5950 (0.0153)	0.5780 (0.0278)	**0.0069**
3	MD	7.19 × 10^–4^ (2.19 × 10^–5^)	7.42 × 10^–4^ (2.79 × 10^–5^)	**0.0028**	0.7189 (0.0157)	0.7416 (0.0242)	**0.0002**
4	RD	4.63 × 10^–4^ (2.63 × 10^–5^)	4.88 × 10^–4^ (3.16 × 10^–5^)	**0.0046**	0.4627 (0.0198)	0.4876 (0.0270)	**0.0006**
5	Trails‐B	56.9375 (15.0575)	77.1389 (30.9222)	**0.0027**	56.9375 (13.2685)	76.9072 (29.9127)	**0.0016**
6	WAIS‐dig	7.3125 (1.8154)	5.7500 (1.9030)	**0.0084**	7.3125 (1.7554)	5.7821 (1.7639)	**0.0071**
7	WCST‐per	55.3125 (11.9372)	55.7941 (22.9502)	0.9227	55.3125 (9.7660)	53.1081 (25.5459)	0.6553
8	LF‐F&A&S	39.6875 (7.5694)	36.3056 (11.2016)	0.2104	39.6875 (6.2409)	36.3959 (10.4273)	0.1655
9	CVLT‐LDFR	12.0000 (2.5298)	9.4444 (2.3354)	**0.0019**	12.0000 (2.4856)	9.3749 (1.9578)	**0.0010**
10	CVLT‐total	53.3750 (10.0391)	45.2778 (11.2596)	**0.0145**	53.3750 (9.8371)	45.2728 (9.6563)	**0.0101**
11	VRT	51.0000 (15.8661)	41.9714 (15.5137)	0.0678	51.0000 (8.5701)	41.3776 (15.3437)	**0.0059**

Note

*p*‐value in bold when *p* < 0.05.

Abbreviations: CVLT‐LDFR, California Verbal Learning Test: Long Delay Free Recall; CVLT‐total, California Verbal Learning Test trial 1–5 total; DVR, Dynamic Vasomotor Reactivity; FA, Fractional Anisotropy; LF‐F&A&S, Letter Fluency parts F & A & S; MD, Mean Diffusivity; RD, Radial Diffusivity; Trails‐B, Trails‐making part B; VRT, Visual Reproduction t‐score delay recall; WAIS‐dig, Wechsler Adult Intelligence Scale with digit span backward; WCST‐per, Wisconsin Card Sorting Test perceptive responses t‐score.

### 3.2 Pairwise correlations between the 11 markers/scores

In order to examine possible pairwise correlations between the 11 markers/scores considered in this study, we show in Table 3 the Pearson correlation estimates between all pairwise combinations (*after* separating out the effects of the covariates of age, gender, and education) for all MP and CS taken together. The *p*‐value of statistical significance of each pairwise correlation is also given in parentheses underneath the correlation estimate. We observe that the DVR physiomarker is only highly correlated with the three DTI biomarkers (marked in bold italics in Table 3 for *p* < 0.01) and weakly correlated with only one neurocognitive test score, viz. the CVLT‐LDFR for episodic memory (0.01 < *p* < 0.05 marked in italics in Table 3). The latter also strongly correlates with the MD biomarker, while the related CVLT‐total score correlates weakly with MD. As expected, the correlation between CVLT‐LDFR and CVLT‐total is strong. The three DTI biomarkers are strongly correlated among themselves and with the Trails‐B score of attention‐executive function. However, the DVR does not correlate significantly with the Trails‐B score. Among the attention‐executive function neurocognitive scores, only two pairs show significant correlation (WCST‐per vs. LF‐F&A&S and Trails‐B vs. WAIS‐dig). Finally, two pairs of attention‐executive versus episodic memory scores correlate significantly (WAIS‐dig vs. VRT and LF‐F&A&S vs. CVLT‐total). The correlations of the DVR with the DTI biomarkers and with the CVLT‐LDFR score are examined further through regression analysis below.

**Table 3:** Pairwise Pearson correlations and *p*‐values between the markers/scores for all CS and MP

Pearson correlation (*p*)	Hemodynamic physiomarker	DTI biomarkers			Attention‐executive function scores				Episodic memory scores	
	DVR	FA	MD (×10^3^)	RD (×10^3^)	Trails‐B	WAIS‐dig	WCST‐per	LF‐F&A&S	CVLT‐LDFR	CVLT‐Total
FA	***0.6107 (<0.001)***									
MD (×10^3^)	***−0.6197 (<0.001)***	***−0.6545 (<0.001)***								
RD (×10^3^)	***−0.6045 (<0.001)***	***−0.8188 (<0.001)***	***0.8744 (<0.001)***							
Trails‐B	−0.2270 (0.106)	***−0.4601 (<0.001)***	***0.5217 (<0.001)***	***0.5450 (<0.001)***						
WAIS‐dig	0.2104 (0.134)	0.1147 (0.417)	−0.1803 (0.200)	−0.1670 (0.236)	*−0.3288 (0.018)*					
WCST‐per	0.0025 (0.986)	−0.0682 (0.631)	0.1553 (0.272)	0.0712 (0.616)	0.2312 (0.099)	−0.2492 (0.075)				
LF‐F&A&S	0.2105 (0.134)	0.1455 (0.3025)	−0.3402 (0.014)	−0.2475 (0.077)	−0.3092 (0.026)	0.2805 (0.044)	***−0.4673 (<0.001)***			
CVLT‐LDFR	*0.2826 (0.043)*	0.0629 (0.657)	***−0.3605 (0.009)***	−0.2302 (0.101)	−0.1536 (0.276)	0.1540 (0.275)	−0.1971 (0.161)	0.2541 (0.069)		
CVLT‐total	0.1904 (0.176)	−0.0071 (0.960)	*−0.3506 (0.011)*	−0.1515 (0.283)	−0.2193 (0.118)	0.2130 (0.129)	−0.1220 (0.389)	*0.3292 (0.018)*	***0.7265 (<0.001)***	
VRT	−0.0762 (0.591)	0.0850 (0.548)	−0.0371 (0.793)	−0.0493 (0.728)	−0.2526 (0.071)	***0.3745 (0.007)***	−0.0934 (0.510)	−0.0366 (0.796)	0.1831 (0.193)	0.0812 (0.566)

Note

All cases with *p* < 0.01 are highlighted in bold italic and with 0.01 < *p *< 0.05 are highlighted in italic.

### 3.3 Linear regression between DVR and the DTI biomarkers or the CVLT‐LDFR score

**Figure 1:**
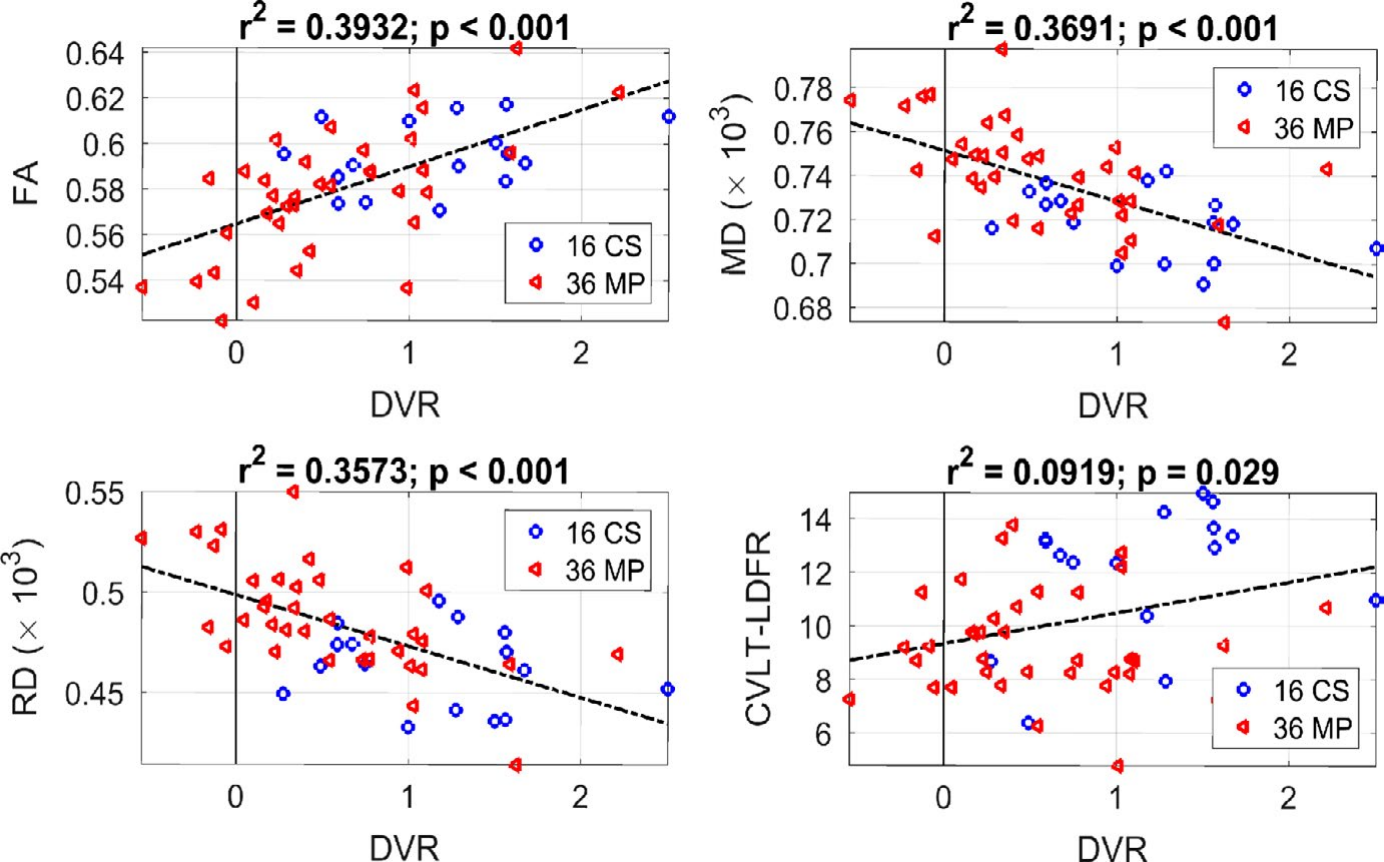
Scatter plots of DVR versus FA (top left), MD (top right), RD (bottom left), and CVLT‐LDFR (bottom right) with estimated regression lines (dotted). The *r* ^2^ and *p*‐values are shown at the top of each plot. The estimated linear regression equations are: FA = 0.025 × DVR + 0.564; MD (×10^3^) = −0.022 × DVR + 0.751; RD (×10^3^) = −0.025 × DVR + 0.499; CVLT‐LDFR = 1.15 × DVR + 9.338

Since the novel DVR physiomarker is the focus of this paper, its relations with the DTI biomarkers and neurocognitive scores are examined further in this section through regression analysis, which is limited to the markers/scores with which significant correlations exist (see Table 3). The scatter plots of DVR versus the three DTI biomarkers (FA, MD, and RD) extracted from the significant voxels and the CVLT‐LDFR neurocognitive score of episodic memory are shown in Figure 1, along with the estimated regression lines. The *r* ^2^ values are substantial (and highly significant with *p* < 0.001) for DVR versus the three DTI biomarkers, but marginal for DVR versus the CVLT‐LDFR score (*r* ^2^ = 0.09 and *p* = 0.029). We note that CVLT‐LDFR is the memory‐related neurocognitive score that shows the strongest correlation with a DTI biomarker, viz. MD (see Table 3).

### 3.4 Associations of DVR with DTI biomarkers in specific brain regions

To provide more insight into the numbers of significant voxels of Fractional Anisotropy (FA), Mean Diffusivity (MD), and Radial Diffusivity (RD) that have been found in various brain regions by our analysis (i.e., the voxels where DTI biomarker values correlate significantly with DVR), we show in Table 4 those numbers of significant voxels and the respective percentages for 48 brain regions (R: right side, L: left side). The highest percentages (>30%) of significant voxels were found in the 22 brain regions highlighted in boldface in Table 4.

**Table 4:** The numbers of significant voxels of Fractional Anisotropy (FA), Mean Diffusivity (MD), and Radial Diffusivity (RD)—that is, correlating significantly with DVR—which were found in 48 brain regions

Brain region	FA voxels	FA (%)	MD voxels	MD (%)	RD voxels	RD (%)
Middle cerebellar peduncle	6	(0.2)	0	(0)	3	(0.1)
Pontine crossing tract	0	(0)	0	(0)	0	(0)
Genu of corpus callosum	0	(0)	393	(20.8)	250	(13.2)
**Body of corpus callosum**	0	(0)	1,302	(39.5)	987	(30)
**Splenium of corpus callosum**	0	(0)	736	(33)	407	(18.2)
Fornix column body	0	(0)	0	(0)	0	(0)
Corticospinal tract R	37	(10.1)	0	(0)	10	(2.7)
Corticospinal tract L	0	(0)	0	(0)	0	(0)
Medial lemniscus R	0	(0)	0	(0)	0	(0)
Medial lemniscus L	0	(0)	0	(0)	0	(0)
Inferior cerebellar peduncle R	0	(0)	0	(0)	0	(0)
Inferior cerebellar peduncle L	0	(0)	0	(0)	0	(0)
Superior cerebellar peduncle R	0	(0)	0	(0)	0	(0)
Superior cerebellar peduncle L	0	(0)	0	(0)	0	(0)
**Cerebral peduncle R**	258	(47.9)	0	(0)	111	(20.6)
Cerebral peduncle L	0	(0)	0	(0)	0	(0)
**Anterior limb of internal capsule R**	359	(48.1)	247	(33.1)	46	(6.2)
**Anterior limb of internal capsule L**	65	(9.1)	184	(25.8)	219	(30.8)
**Posterior limb of internal capsule R**	508	(52.3)	29	(3)	205	(21.1)
Posterior limb of internal capsule L	0	(0)	85	(9)	227	(24.1)
**Retrolenticular part internal capsule R**	16	(2.1)	173	(23.1)	23	(3.1)
Retrolenticular part internal capsule L	2	(0.3)	390	(54.5)	374	(52.3)
Anterior corona radiata R	75	(4.8)	332	(21.3)	0	(0)
Anterior corona radiata L	27	(1.6)	475	(28.9)	299	(18.2)
Superior corona radiata R	49	(3.6)	158	(11.7)	0	(0)
Superior corona radiata L	30	(2)	389	(26.1)	411	(27.6)
**Posterior corona radiata R**	0	(0)	375	(43.8)	384	(44.8)
**Posterior corona radiata L**	0	(0)	300	(41.0)	317	(43.3)
**Posterior thalamic radiation R**	0	(0)	389	(40.4)	260	(27.0)
**Posterior thalamic radiation L**	150	(17.2)	345	(39.6)	387	(44.4)
**Sagittal stratum R**	5	(0.8)	291	(45.7)	101	(15.9)
Sagittal stratum L	0	(0)	47	(7.4)	172	(27.1)
**External capsule R**	317	(22)	478	(33.2)	0	(0)
**External capsule L**	162	(11.4)	461	(32.4)	522	(36.6)
Cingulum cingulate gyrus R	0	(0)	0	(0)	46	(22.0)
**Cingulum cingulate gyrus L**	0	(0)	17	(7.6)	88	(39.3)
Cingulum hippocampus R	0	(0)	0	(0)	0	(0)
Cingulum hippocampus L	0	(0)	0	(0)	0	(0)
**Fornix cres Stria terminalis R**	0	(0)	106	(32.9)	7	(2.2)
**Fornix cres Stria terminalis L**	14	(4.1)	88	(25.9)	165	(48.5)
Superior longitudinal fasciculus R	0	(0)	176	(9.8)	106	(5.9)
**Superior longitudinal fasciculus L**	31	(1.9)	401	(24.3)	601	(36.5)
Superior fronto‐ occipital fasciculus R	13	(11.5)	5	(4.4)	0	(0)
**Superior fronto‐occipital fasciculus L**	0	(0)	12	(13.2)	34	(37.4)
**Uncinate fasciculus R**	42	(68.9)	20	(32.8)	0	(0)
Uncinate fasciculus L	0	(0)	0	(0)	0	(0)
**Tapetum of corpus callosum R**	0	(0)	64	(73.6)	49	(56.3)
**Tapetum of corpus callosum L**	0	(0)	55	(70.5)	45	(57.7)
Whole mask voxels	2,637	(2.1)	13,519	(10.5)	10,696	(8.3)

Note

The numbers in parentheses represent the percentage of significant voxels in each region.

## 4 DISCUSSION

This study sought to compare a novel model‐based “physiomarker” of Dynamic Vasomotor Reactivity to CO2 (DVR) that has been recently shown to delineate amnestic MCI patients from age‐matched controls without cognitive impairment (Marmarelis et al., 2017) with the widely used DTI biomarkers of Fractional Anisotropy (FA), Mean Diffusivity (MD), and Radial Diffusivity (RD), as well as with five neurocognitive assessment scores of executive function and episodic memory. The selection of these markers/scores is based on the fact that each of them can delineate (*p* < 0.01) amnestic MCI patients from age‐matched controls (see Table 2) after accounting for the effects of the covariates of age, gender, and education. We note that the efficacy of the DTI biomarkers is assessed over the “significant” voxels where significant correlation exists between the DTI biomarkers and the DVR physiomarker.

The main finding of this study is that significant pairwise Pearson correlations exist between the DVR physiomarker and the three DTI biomarkers of FA, MD, and RD (*p* < 0.001), as well as between the DTI biomarkers and the Trails‐B neurocognitive test score (see Table 3). Furthermore, we found significant regression lines (associations) between DVR and the three DTI biomarkers, as well as the CVLT‐LDFR neurocognitive score that is related to episodic memory (see Figure 1). We also found that only the MD DTI biomarker correlates significantly with the CVLT‐LDFR and CVLT‐total neurocognitive scores, and only four pairwise correlations of neurocognitive scores are significant (see Table 3).

### 4.1 Diagnostic equivalence between the DVR physiomarker and the DTI biomarkers

Our results suggest that the model‐based DVR physiomarker of metabolic cerebrovascular regulation provides delineation between MCI patients and cognitively normal controls (*p* < 0.01) comparable to the performance of the DTI biomarkers of FA, MD, and RD (as well as comparable to the performance of the most efficacious neurocognitive tests related to executive function and episodic memory). We note that the DTI biomarkers measure the amount of water diffusion in white matter (WM) fiber tracts that are physically restricted by axonal membranes and myelin (Mori & Zhang, 2006) and their values are thought to provide measures of the integrity of the WM neuronal fibers. Since the major WM fiber tracts that may be susceptible to cerebral hypoperfusion and/or ischemia are primarily found in the deep and periventricular brain regions, such as the corpus callosum, the corona radiata, the internal capsule, the external capsule, and the superior longitudinal fasciculus (Moody et al., 1990), it is notable that these brain regions are among those listed in Table 4 as having large percentages of significant voxels with high correlation between DTI biomarkers and the DVR physiomarker.

Cognitive function performance has been correlated with DTI biomarkers from global and regional WM fiber tracts in previous studies: processing speed and executive function (Charlton et al., 2006), processing speed and global cognition (Vernooij et al., 2009), and executive function (Tarumi et al., 2015)—while it was also shown that total brain volume of WM or WM hyperintensities were not correlated to cognitive performance. We note that, although the DTI biomarkers in global WM (quantifying the integrity of the WM neuronal fibers) were shown to correlate with executive function performance in MCI patients and cognitively normal controls alike, the MCI patients with lower executive function performance showed statistically similar levels of WM neuronal fiber integrity with cognitively normal controls (Tarumi et al., 2015). This intriguing discrepancy between the group statistical comparison and regression analysis may be due to the limited cohort size of these studies or to the multifactorial nature of cognitive impairment that includes amyloid deposition, hypometabolism, and cardiovascular/cerebrovascular dysregulation, among many others, which may influence cognitive function independently of defects in WM structural integrity (Arnaiz et al., 2001; Jack et al., 2009; Marmarelis et al., 2017; Tarumi et al., 2015). In the present study, when only the “significant” voxels of DTI brain images (i.e., those with significant correlation with DVR) were used for group statistical comparison, the DTI biomarkers delineated MCI patients from controls. The DVR physiomarker (being a global cerebrovascular measure for each subject) also achieves significant delineation of MCI patients from controls and exhibits significant pairwise Pearson correlations with the DTI biomarkers and the CVLT‐LDFR score (see Table 3), as well as significant associations via regression analysis (see Figure 1). Note that the CVLT‐LDFR test relates to episodic memory, while none of the neurocognitive tests related to executive function had significant pairwise correlation with DVR (see Table 3). This suggests that DVR may be related more to memory deficits than executive dysfunction.

### 4.2 Results from specific brain regions

**Figure 2:**
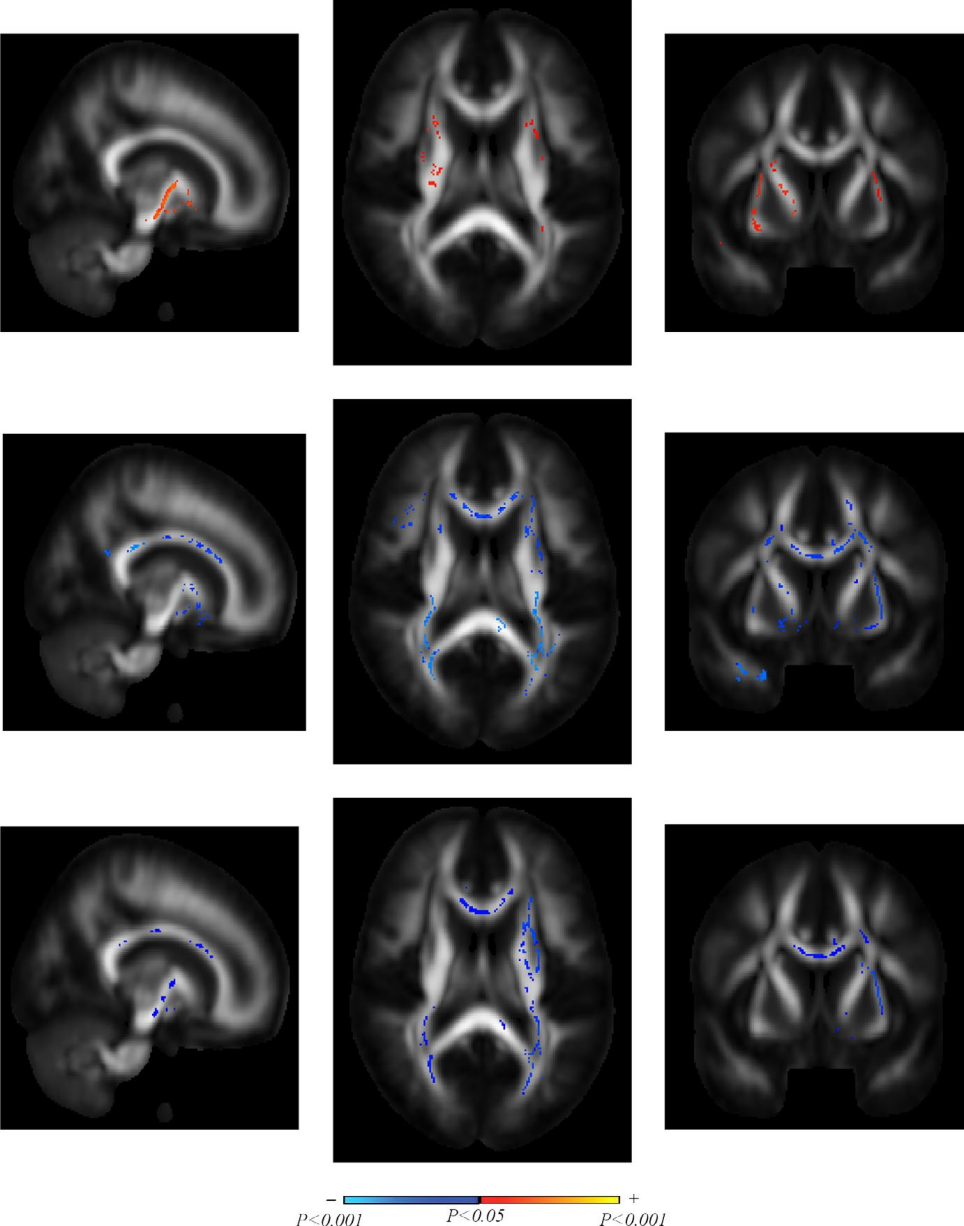
Illustrative example of voxel‐wise analysis of the correlation between DVR and localized DTI metrics in regions of brain white matter (computed from the significant voxels only): sagittal (left), axial (middle), and coronal (right) views, for FA (top panels), MD (middle panels), and RD (bottom panels). Color inside the brain maps indicates the areas that are significantly correlated with DVR (according to the color bar at the bottom of the Figure)

Using the *significant* voxels of DTI brain images (i.e., the voxels that show significant correlation between DVR and DTI values) over all subjects, we found that 22 of 48 analyzed brain regions contained more than 30% significant voxels (see Table 4). An illustrative example is shown in Figure 2 that demonstrates the localized correlations of DVR with DTI metrics in some brain regions. Among the 22 brain regions with high percentages (>30%) of significant voxels, we note the very high percentages (>50%) in the *right posterior limb of the internal capsule*, the *left retrolenticular part of the internal capsule*, the *right uncinate fasciculus,* and the *tapetum of corpus callosum* (right and left). The latter has the highest percentage of significant voxels (>70%) with respect to MD values and represents—like the *uncinate fasciculus*—a WM association tract in the human brain that connects parts of the limbic system (such as the *hippocampus* and *amygdala* in the temporal lobe) with parts of the frontal cortex (such as the *orbitofrontal cortex*). The finding of high percentage of significant voxels in the *right posterior limb* and *the left retrolenticular part of the internal capsule* is reasonable, because these parts of the internal capsule are supplied to a large extent by the lenticulostriate arteries (Djulejik et al., 2016), which are branches of the middle cerebral artery where the transcranial Doppler (TCD) measurements of cerebral blood flow velocity were made, from which the hemodynamic model that generates the DVR is derived. We also note that the other parts of the internal capsule are supplied by other cerebral arteries (viz. the lower and anterior part is perfused by the perforators of the anterior cerebral artery, and the genu is perfused by the perforators of the internal carotid and anterior choroidal arteries), which may not be affected directly by the TCD measurements that yield the DVR physiomarker. The regional differences in brain perfusion and DTI abnormalities, and their correlations with neurocognitive function, need to be further studied in the context of neurocognitive disease (for instance, abnormalities in the *uncinate fasciculus* were found to correlate with executive dysfunction in patients with left temporal lobe epilepsy [Diao et al., 2015]).

The findings of this study lead to the conclusion that the model‐based cerebral hemodynamic index DVR, which constitutes a physiomarker capable of delineating amnestic MCI patients from cognitively normal controls (*p* < 0.01), correlates (*p* < 0.001) with the MRI‐DTI biomarkers in various brain regions—especially with the ones perfused by the perforating arteries of the middle cerebral artery (which is the location of the TCD measurement of cerebral blood flow velocity from which the model‐based DVR is derived), as well as with the scores of the CVLT‐LDFR neurocognitive test that is related to episodic memory. These results corroborate the view that the DTI biomarkers may be influenced by dysfunction of brain microcirculation, and that DVR is a useful diagnostic physiomarker in conjunction with DTI biomarkers in patients with MCI.

## CONFLICT OF INTEREST

The authors have no conflict of interest to report.

## Data Availability

The data that support the findings of this study are available from the corresponding author upon reasonable request.
